# Identification and Characterization of Three Novel B-Cell Epitopes in African Swine Fever Virus p22 Protein

**DOI:** 10.3390/microorganisms13122666

**Published:** 2025-11-24

**Authors:** Zehui Li, Jingming Zhou, Yumei Chen, Hongliang Liu, Yanhua Qi, Chao Liang, Xifang Zhu, Enping Liu, Sixuan Wu, Peiyang Ding, Aiping Wang

**Affiliations:** 1 Longhu Laboratory of Advanced Immunology, Zhengzhou 450046, China; lizehui2091@163.com (Z.L.);; 2 Henan Provincial Key Laboratory of Immunological Biology, Zhengzhou 450001, China; 3 School of Life Science, Zhengzhou University, Zhengzhou 450001, China

**Keywords:** African swine fever virus p22, recombinant expression, monoclonal antibodies, novel B-cell epitopes, conservation analysis

## Abstract

African swine fever virus (ASFV) is a highly contagious pathogen causing African swine fever in wild boars, warthogs and domestic pigs. The disease leads tosubstantial economic losses to the global pork industry and poses a grave threat to biodiversity. The early-encoded structural protein p22, owing to its immunodominant characteristics and high conservation across most genotypes, represents a promising diagnostic target and subunit vaccine candidate. In this study, the soluble extracellular domain of p22 protein (aa 30–177) was successfully expressed and purified, yielding 1.220 g/L. Eleven strains of monoclonal antibodies against p22 were generated, with four selected for B-cell epitope screening. Bioinformatic prediction-guided design was employed to generate overlapping truncations and peptides for epitope mapping. Based on those strategies, three novel linear B-cell epitopes were identified to be ^30^KKQQPPKK^37^, ^130^WGTDDCTG^137^ and ^150^YVYNNPHH^157^ by monoclonal antibodies. Sequence alignment across ASFV isolates revealed 100% evolutionary conservation in genotypes I/II, with minor variation in genotypes IV/VIII/XX/XXII. This study provided valuable data for broadening the ASFV antigen spectrum and identifying immunological targets for subunit vaccine formulation strategies.

## 1. Introduction

African swine fever (ASF), caused by African swine fever virus (ASFV), is a devastating and highly contagious global disease characterized by hemorrhagic fever, imposing substantial socio-economic burdens, inflicting massive losses to the domestic pig industry and threatening food security and biodiversity [1].

Transmission occurs through complex epidemiological cycles [2,3] involving direct contact, soft ticks (known as biological vectors and reservoirs [4]), habitat contamination and indirect contact via contaminated materials such as tissues, uncooked meat and anthropogenic activities during pork trade or transportation [5].

In acute ASF infections, clinical symptoms typically present as respiratory distress, lethargy or anorexia, with high fever, centripetal cyanosis, pulmonary edema and multifocal hemorrhage [6], accompanied by hematological abnormalities such as thrombocytopenia, neutropenia and lymphopenia [5]. Subacute and chronic infection are characterized by splenomegaly, lymphadenectasis, peritoneal or pericardial effusion, arthritis, skin lesions, renal disturbance and sow abortion [7,8].

The first escape of ASF prevalence, which was once confined to African continent, was described in Portugal in 1957. It subsequently spread along the Iberian Peninsula to European countries and across the Atlantic from 1960 to 1995 [9,10,11]. The second wave of the disease was confirmed in Georgia [12], gradually spreading to other neighboring countries including Azerbaijan, Russia and Belarus [9]. From 2012 to 2018, ASF burst into an outbreak, penetrating the European Union border to Eastern European countries including Lithuania, Latvia, Estonia, Hungary, the Czech Republic, Romania and Belgium [10], and eventually, it reached Asia. In 2018, ASF was first reported in China [13], followed by its appearance in Russia, Mongolia, Vietnam, Cambodia, North Korea, Laos, Myanmar, the Philippines, South Korea, Timor-Leste and Indonesia [14].

Effective control of ASF critically depends on accurate and timely diagnosis. Serological assays detecting ASFV-specific antibodies are vital tools for surveillance and confirming infection cases. Consequently, identifying highly immunogenic viral antigens is essential for developing robust serological tests.

The p22 protein, an early-expressed structural protein [15,16], is involved in ASFV membrane precursor formation [17,18,19,20], exhibiting strong immunodominance during the humoral immune response against ASFV [21]. Furthermore, p22 demonstrates high conservation across diverse ASFV strains, making it a promising diagnostic target for broad serological detection [22,23,24].

Here, we integrated bioinformatics-based and experimental approaches to identify and validate novel B-cell epitopes within the p22 protein. Three epitopes were characterized by newly generated monoclonal antibodies (mAbs). This study aims to systematically characterize the immunogenic landscape of p22 and extend the antigen spectrum of ASFV, thereby furnishing an experimental dataset for the development of multi-epitope-combined diagnostic assays for ASFV.

## 2. Materials and Methods

### 2.1. Cells, Serum and Reagents

SP2/0 myeloma cells and human embryonic kidney 293T (HEK293T) cells were maintained in Roswell Park Memorial Institute 1640 (RPMI 1640, Gibco, Middleton, WI, USA) and Dulbecco’s modified Eagle’s medium (DMEM, Gibco, Middleton, WI, USA), respectively, with 10% (*v*/*v*) fetal bovine serum (FBS, Oricell, Shanghai, China). pET-32a plasmid, *Escherichia coli* (*E. coli*) DH5α and Rosetta (DE3) were obtained from Novagen (Darmstadt, Germany).

jetPRIME^®^ transfection reagent (catalog no. 114-01), Ni Sepharose^TM^ Excel resin (catalog no. 17-3712-02) and PEG 1500 (catalog no. 10783641001) were purchased from Polyplus-transfection^®^ (New York, NY, USA), GE Health Care (Chicago, IL, USA) and Roche (Basel, Switzerland), respectively. *Nco*I-HF and *Bam*HI-HF restriction enzymes were purchased from New England Biolabs (NEB, Ipswich, MA, USA). T4 DNA Ligase and Premix Ex Taq^®^ DNA Polymerase were purchased from Takara Biomedical Technology Corporation, Ltd. (Beijing, China). IPTG, Ampicillin, a BCA Protein Assay Kit, DAPI, HRP-conjugated anti-His Tag mAb and an AEC Peroxidase Substrate Kit were purchased from Sorlarbio Life Sciences (Beijing, China). HRP-conjugated goat anti-mouse antibody, HRP-conjugated goat anti-swine antibody and Dylight 594 labeled goat anti-mouse IgG were purchased from Abbkine Scientific Corporation, Ltd. (Wuhan, China). An eECL Western blot kit was purchased from CWbiotech Corporation, Ltd. (Taizhou, Jiangsu, China). A mouse mAb subtype identification kit was purchased from Proteintech Corporation, Ltd. (Wuhan, China). The ASFV-positive serum were purchased from National Center for Veterinary Culture Collection of China (Lot: 202112).

### 2.2. Bioinformatical Analysis

The KP177R gene (Genbank Accession: MK128995) was optimized as the codon bias of *E. coli* required and synthesized by Sangon Biotech Corporation, Ltd. (Shanghai, China). The transmembrane domain of p22 was predicted by the online service TMHMM 2.0 (https://services.healthtech.dtu.dk/services/TMHMM-2.0/, accessed on 5 September 2024).

B-cell epitopes of p22 were predicted via different bioinformatic algorithms and tools including Kolaskar-Tongaonkar, Parker, Chou-Fasman, Karplus-Schulz, Emini and BepiPred in IEDB database (http://tools.iedb.org/bcell/, accessed on 5 September 2024).

The conservation of amino acid sequences was analyzed using MUSCLE alignment (Version 3.8.21) in the Jalview software (Version 2.11.5.0). The three-dimensional structure of p22 was predicted by I-TASSER (https://zhanglab.ccmb.med.umich.edu, accessed on 22 September 2024) de novo modeling and SWISS-MODEL homologous modeling (https://swissmodel.expasy.org/interactive, accessed on 25 August 2022). The identified B-cell epitopes were visualized on the model by PyMOL software (Version 3.1.3.1).

### 2.3. Preparation of Recombinant p22 Protein

The pair of primers shown in Table 1 was designed by SnapGene (Version 5.3) to amplify the extra-envelope region (30–177 aa) of the KP177R gene. The following thermal programs were as follows: one cycle at 95 °C for 3 min; 32 cycles at 95 °C for 30 s, 55 °C for 30 s and 72 °C for 45 s; one cycle at 72 °C for 10 min and 16 °C for final cooling. The PCR product with restriction sites was then cleaved by *Bam*H I and *Xho* I enzymes, cloned into the vector pET-32a and transformed into *Escherichia coli* DH5*α* competent cells. The recombinant expression plasmid pET-32a-KP177R was extracted from positive clones confirmed by PCR and sequencing. Similarly, the recombinant plasmid pcDNA3.1-p22-His used for the immunofluorescence assay was also constructed in accordance with the protocol above. The recombinant plasmid pET-32a-KP177R was further transformed into *Rosetta* (DE3) competent cells, and positive clones were selected for expression. An aliquot of 2 mL of overnight *Rosetta* (DE3) culture was expanded into 200 mL of LB medium containing 0.1% Ampicillin. To achieve the maximum amount of soluble expression of recombinant p22, a series of expressing conditions were optimized by orthogonal test, including temperature for expression, concentration of IPTG and incubation time (Table 2). The results were performed by 12% SDS-PAGE (Mini-Protean, PowerPac and Bio-Rad Gel Doc XR Imager, Bio-Rad, Hercules, CA, USA). The intensity of gel bands was analyzed by ImageJ (Version 1.53q) and the results were used to compare the protein quantity in the forms of soluble and inclusion bodies under different orthogonal conditions.

After expression, the cell pellets were harvested at 8000× *g* centrifugation for 5 min, then resuspended by Tris-HCl (20 mmol/L, containing 150 mmol/L NaCl, pH 8.0) and ultrasonicated on ice for 10~20 min. Then the suspension was centrifuged for another 15 min at 12,000× *g* to separate the soluble protein in the supernatant and the inclusion bodies in the precipitate.

Soluble p22 protein in supernatant with N-terminal Trx-His fusion was purified by Ni Sepharose^®^ Excel column in 50 mM Tris-HCl buffer, and the target protein was competitively eluted with different imidazole concentration gradients ranging from 10 mM to 500 mM. The eluted fractions were monitored by UV absorbance value at 280 nm, collected and analyzed by SDS-PAGE. The concentration of purified protein was determined by BCA Protein Assay Kit and stored at −20 °C for use.

### 2.4. Western Blot

The recombinant p22 protein was determined byWestern blot as described [25]. In brief, SDS-PAGE was used for protein separation and target band blotting was processed with Trans-Blot^®^ SD Semi-Dry Electrophoretic Transfer Cell (Bio-Rad, USA) from gels to PVDF membranes (0.45 μm, Millipore, Burlington, MA, USA). The membranes were blocked with 5% skimmed milk diluted in PBST (0.01 mmol/L phosphate-buffered saline with 0.05% Tween-20) at 4 °C overnight. Then the membranes were incubated with tag-specific antibodies and the ASFV-positive serum separately for 1 h. After five rounds of washing, HRP-conjugated goat anti-mouse/swine IgG (1:5000) was used as the secondary antibody. The PVDF membranes were incubated for another 1 h and washed five times. The enzyme–substrate reaction was exposed by the Tanon 5200 automatic imaging system (Shanghai, China) with the eECL Western blot kit.

### 2.5. Preparation of p22-Specific Monoclonal Antibodies

**Figure 1:**
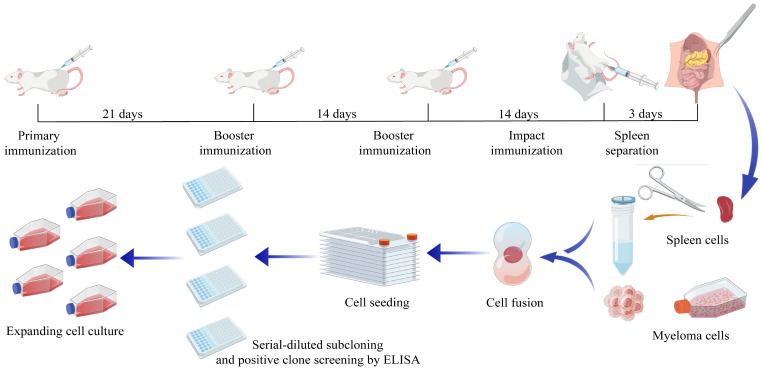
Procedure of mouse immunization and preparation of monoclonal antibodies.

p22-specific mAbs were produced as described [26] (Figure 1). Three BALB/c mice aged 6–8 weeks were subcutaneously injected in the back with 50 μg of recombinant p22 protein emulsified with Freund’s complete adjuvant (Sigma-Aldrich, Shanghai, China). Two rounds of booster immunizations, containing 20 μg of protein emulsified with Freund’s incomplete adjuvant, were carried out biweekly after the primary immunization. The titer of p22-specific antibody was detected through indirect ELISA and mice with higher titer were given an intraperitoneal impact immunization with 50 μg of recombinant protein solution in PBS.

After 3–4 days, the mice were sacrificed and splenocytes were aseptically harvested and subsequently used for cell fusion. In detail, the spleens of the immunized mice were separated, gently ground through a 200-mesh nylon cell strainer, and diluted into a single-cell suspension with GNK solution (PBS with 5 mM glucose and 14 mM phenol red, pH 7.4). After being centrifuged, myeloma cells were mixed with splenocytes at a ratio of 1:5 to 1:8. Then PEG 1500 was slowly added dropwise to the cell mixture, maintaining at 37 °C in a water bath. After 90 s, the reaction was terminated with GNK solution, followed by another incubation for 5 min. Cells were finally diluted with culture medium and seeded into 96-well plates.

10–12 days after cell fusion, hybridoma cells were screened through indirect ELISA. Positive hybridoma cells were subcloned with multiple gradients of dilution on 96-well plates. Monoclonal hybridoma cell lines with high antibody titer and considerable stability were further confirmed by an immunofluorescence assay (IFA) using recombinant p22 protein transiently expressed in HEK293T cells. In order to obtain large scale of mAbs, ascites was induced by liquid paraffin together with monoclonal hybridoma cells and purified with a caprylic acid–ammonium sulfate precipitation.

### 2.6. Indirect ELISA

The antibody titer of p22-immunized mice and monoclonal hybridoma cells was determined by indirect ELISA described as follows [27]: 96-well microplates were coated with recombinant p22 protein (2 μg/mL) and the supernatant of the pET-32a-expressed product as a negative control, diluted in carbonate/bicarbonate buffer (CBS, 0.05 mmol/L, pH 9.6), and incubated overnight at 4 °C. Then the microplates were washed three times, and 5% skimmed milk was used to block uncombined active sites for 1 h at 37 °C. The serum of immunized mice was 2-fold diluted at the beginning ratio of 1:200 in triplicate and incubated for 1 h at 37 °C. After five washes, HRP-conjugated goat anti-mouse IgG (1:5000) was trapped onto the solid phase for another incubation in the same condition. The enzyme–substrate reaction was performed with tetramethyl benzidine (TMB, 5 mmol/L) as substrate and stopped after 5 min by 2 mol/L H_2_SO_4_. The optical density (OD) at 450 nm was read by SpectraMax i3x Platform (Molecular Devices, Shanghai, China). The orbital serum of sacrificed mice and the serum of mice immunized with PBS were used as the positive control and negative control, respectively.

### 2.7. Immunofluorescence Assay

Immunofluorescence assays (IFA) were used to further confirm the reactivity of anti-p22 mAbs with the recombinant p22 protein expressed in the eukaryotic system. HEK293T cells were seeded in a poly-L-lysine pretreated 96-well plate with a density of 5 × 10^4^ cells per well and cultured at 37 °C with 5% CO_2_ overnight until the confluence reached 70–90%. The recombinant plasmid pcDNA3.1-p22-His was transfected into 293T by jetPRIME^®^ transfection reagent. After 48 h, the transfected 293T cells were fixed with paraformaldehyde for 15 min and then permeabilized with 5% TritonX-100 for 20–30 min. After blocking with 5% skimmed milk for 1 h, the fixed cells were incubated with specific antibodies for 1 h at 37 °C. Alexa fluor 488-labeled goat anti-mouse IgG were used as secondary antibodies for another 1 h incubation, with a dilution of 1:200. DAPI was used for DNA-staining, and the immunofluorescent reaction was captured by an inverted fluorescence microscope.

### 2.8. Construction and Expression of Truncated p22 Segments

Based on the bioinformatical prediction sites in Section 2.2, three overlapping truncations (S1/S2/S3) covering the whole extra-envelope region of the KP177R gene were designed. Three pairs of primers were synthesized by Sangon Biotech Corporation, Ltd. (Shanghai, China) for the amplification of three truncations.

The amplified fragments were cloned into pE-SUMO vector and the *Rosetta* (DE3) containing recombinant plasmids (pET-SUMO-S1/S2/S3), which were induced by 0.5 mmol/L IPTG at 37 °C for truncated-protein expression. After harvest, sonication and centrifugation, the supernatant of expression products was collected for indirect ELISA and Dot-blot analysis.

### 2.9. Dot-Blot

Dot-blot was performed to identify regions or peptides recognized by p22-specific mAbs [28]. The nitrocellulose membrane was cut into strips of suitable size and soaked in PBS for 10 min. After being air-dried at room temperature, the strips were coated with truncated protein or BSA-conjugated peptides in the forms of dots with a diameter of 4 mm. The dried dots were blocked with 5% skimmed milk for 2 h at 37 °C and incubated with mAbs and HRP-conjugated goat-anti mouse mAbs (1:5000) in turn. The reaction was finally performed with an AEC Peroxidase Substrate Kit.

### 2.10. Peptide Design and Synthesis

According to the reaction of truncated protein with mAbs, peptides with an offset of 8 amino acids covering the epitope locations predicted by bioinformatic tools were synthesized for the identification of B-cell epitopes. A cysteine residue was added to the N-terminus of each peptide for conjugation with BSA.

The preparation of peptide–BSA conjugates was described as follows: 2 mg sulfo-SMCC was dissolved in 100 μL DMSO and added to 8 mg BSA dissolved in 1 mL conjugating buffer (0.1 M phosphate buffer containing 0.15 M NaCl and 1 μM EDTA, pH 7.2). The mixture was stirred at RT for 30 min and dialyzed for 12 h. A total of 10 μg peptide was added to react with 100 μg SMCC-BSA in 40 μL conjugating buffer for 4 h in 4 °C. The peptide–BSA conjugate was diluted to 1 mg/mL for Dot-blot assay.

### 2.11. Statistical Analysis

The groups of orthogonal experiments were set up by SPSS software (Version 25.0, https://www.ibm.com/spss, accessed on 29 October 2024). All the data in the quantitative graphics of ELISA-formed lines, heat maps and histograms were constructed by GraphPad Prism 8.0 (https://www.graphpad.com/) with three experimental replicates and shown as means ± SD. Multiple comparisons for observed means were processed by two-way ANOVA in GraphPad, and the number of asterisks represented the degree of significance with respect to *p* values as follows: * *p* < 0.05; ** *p* < 0.01; *** *p* < 0.001; **** *p* < 0.0001.

## 3. Results

### 3.1. Expression, Identification and Purification of Recombinant p22 Protein

**Figure 2:**
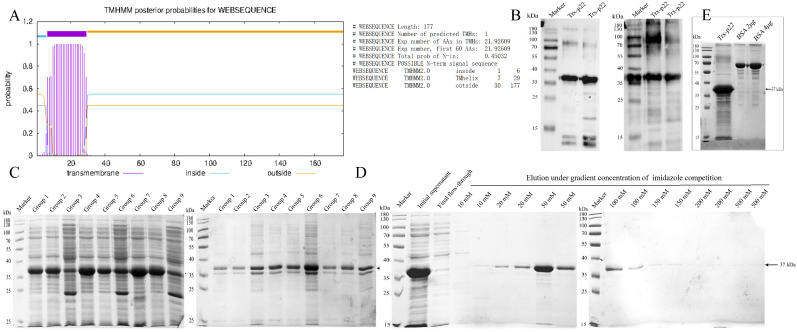
Expression, identification and purification of recombinant p22 protein. (**A**) Prediction of transmembrane domain of p22; (**B**) Identification of p22 recombinant protein with tag-specific antibodies and the ASFV-positive serum by Western blot, separately; (**C**) Supernatant and precipitation of p22 lysate under different orthogonal test conditions; (**D**) Purification of p22 recombinant protein using Ni-NTA affinity chromatography; (**E**) Relative quantification benchmarked by BSA.

The extracellular and transmembrane domains of p22 probabilities were calculated by TMHMM (Figure 2A), and 88–531 bp of KP177R gene was cloned into pET-32a vector for recombinant expression. The extracellular p22 protein fused with the His-Trx tag was identified to be 37 kDa by Western blot. The results showed that the reactivity of recombinant p22 with anti-His mAbs and ASFV-positive serum (Figure 2B) was well-preserved.

The expressing condition was optimized by the orthogonal test shown in Table 2, and the results were performed by SDS-PAGE in Figure 2C. The maximum soluble amount of recombinant protein was expressed in group 7, which was induced by 0.8 mM IPTG in 16 °C for 16 h (Table 3). The optimized recombinant protein yield was determined to be 1.220 g/L, which wascalculated by relative quantitation with BSA in ImageJ software (Version 1.53q) (Figure 2E).

For Ni-NTA affinity chromatography, the His-Trx-p22 fusion was almost fully combined with the medium and was gradually competitively eluted by gradient concentration of imidazole (Figure 2D). The fraction with the highest purity of the target protein, eluted by 50–100 mM imidazole, was then collected for ultrafiltration and immunization. The concentration of purified protein was determined to be 0.757 mg/mL by BCA Protein Assay Kit (Supplementary Figure S1A).

### 3.2. Preparation and Characterization of p22-Specific Monoclonal Antibodies

**Figure 3:**
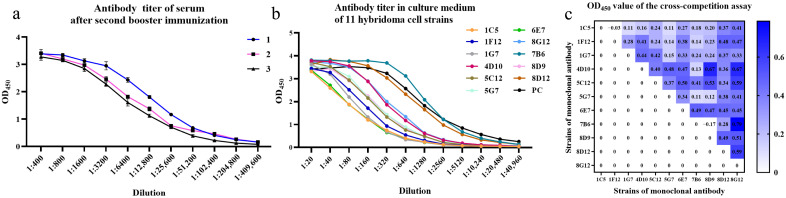
Determination of antibody titers in immunized-mice serum and hybridoma cell lines. (**a**) Titer of serum antibodies after mice were immunized by p22 recombinant protein; (**b**) Titer of antibodies in hybridoma cell culture medium; (**c**) Heat map of cross-competition assay for monoclonal antibody identification.

Following the immunization procedure described in 2.4, the serum antibody titers of three BALB/c mice were all over 1:2.048 × 10^5^ (Figure 3a). Eleven strains of hybridoma cell secreting p22-specific antibodies were successfully isolated through subcloning. The antibody titers in cell culture ranged from 1:5.12 × 10^3^ to 1:4.096 × 10^4^ (Figure 3b). The cross-competition assay was used for the screening of repetitive antibody clones with identical epitope specificity. As shown in Figure 3c, mAbs were divided into three clusters, 1C5-1F12-1G7-5G7-7B6-8D9, 4D10-7B6, and 5C12-8D12. Each group revealed mutually exclusive binding patterns, which suggested the same or proximal epitope recognition. Notably, mAb 8G12 presented as non-competitive with other antibodies, indicating the recognition of another epitope distinct from those targets above.

**Figure 4:**
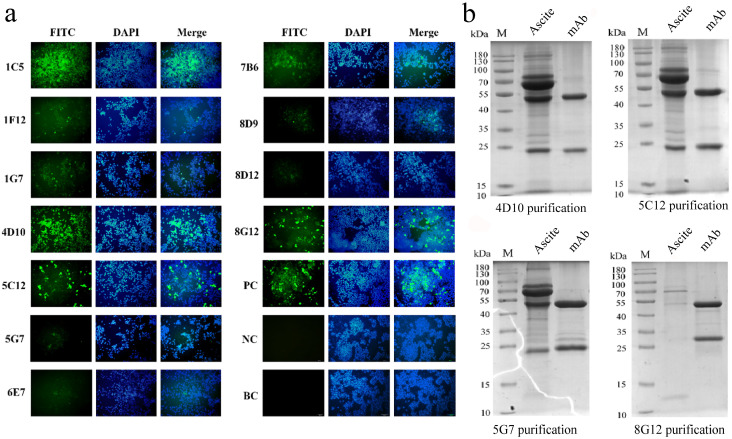
Identification and purification of p22-specific monoclonal antibodies. (**a**) Identification of reactivity between monoclonal antibodies and eukaryotic-expressed p22 by IFA; (**b**) identification of monoclonal antibodies before and after purification by SDS-PAGE.

The HEK293T cells transfected with pcDNA3.1-p22-His were fixed after 48 h expression and co-incubated with mAbs for IFA. Strong reactivity was shown by four strains of mAbs including 1C5, 4D10, 5C12 and 8G12, while a weaker response was shown by other strains (Figure 4a). Since sharing the same epitope, 1C5 was replaced by 5G7 during subsequent study (mentioned in later discussion).

**Figure 5:**
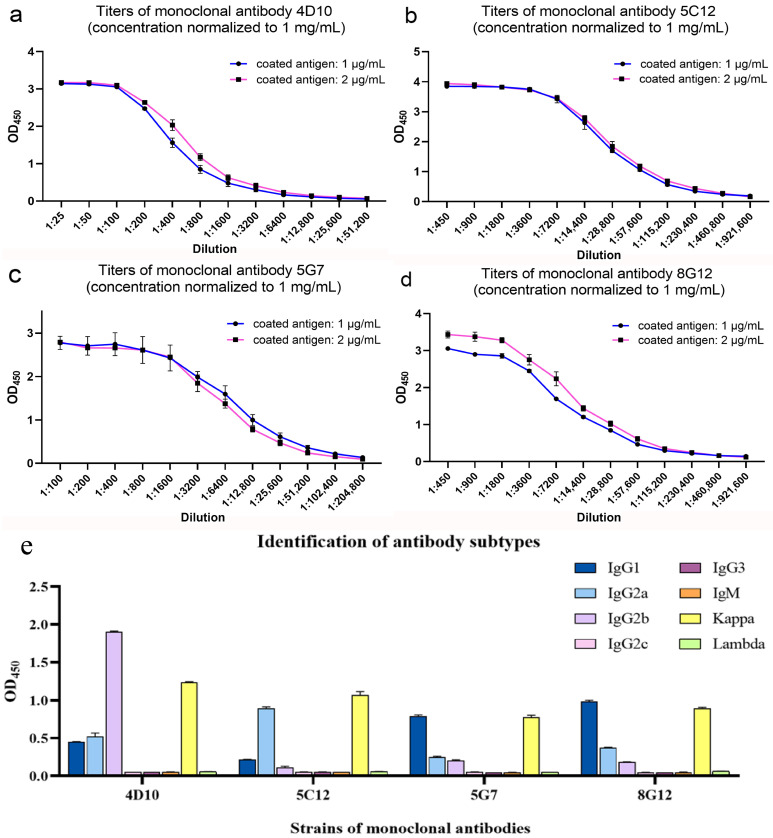
Characteristics of purified monoclonal antibodies. (**a**–**d**) Determination of titers of purified monoclonal antibodies; (**e**) identification of monoclonal antibody subtypes.

After purification, the characteristics of mAbs were identified by SDS-PAGE (Figure 4b) and ELISA. The concentration of mAbs was calculated via BCA Protein Assay Kit (Supplementary Figure S1B) and normalized to be 1 mg/mL. As shown in Figure 5a–d, the titers of mAb 4D10, 5C12, 5G7 and 8G12 reached 1:5.12 × 10^4^, 1:9.216 × 10^5^, 1:2.048 × 10^5^ and 1:9.216 × 10^5^, respectively. The affinity constant of these mAbs was determined to be over 10^7^ L/mol as a previous article described [29] (Supplementary Figure S2). The subtype of these mAbs was determined by a commercial kit and shown in Figure 5e.

### 3.3. Construction and Expression of Truncated p22 Protein

**Figure 6:**
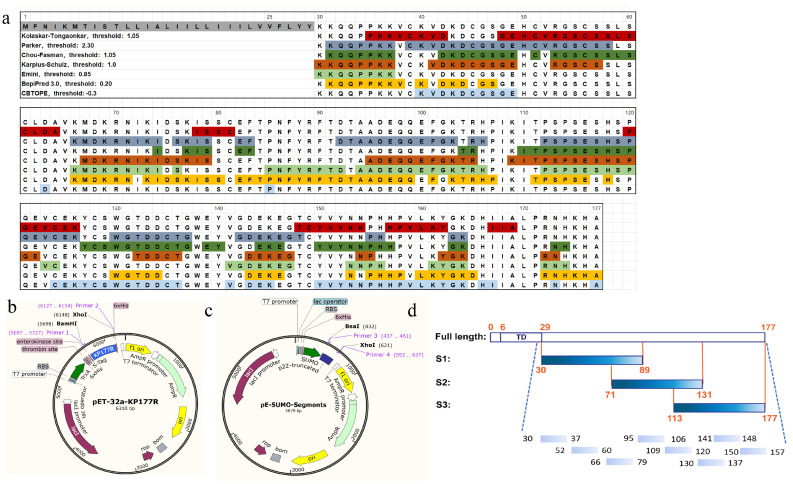
B-cell epitope prediction by bioinformatic algorithms and schematic diagram of truncated p22 protein. (**a**) B-cell epitope prediction by multiple bioinformatic algorithms; (**b**) schematic diagram of recombinant plasmid pET-32a-KP177R; (**c**) schematic diagram of the recombinant plasmids of pE-SUMO-p22-truncated; (**d**) schematic diagram of truncated p22 protein.

According to the bioinformatical epitope prediction generated in Figure 6a, the consistent regions demonstrated by multiple algorithmic analysis were retained. Three truncated segments, including S1 (aa 30–89), S2 (aa 71–131) and S3 (aa 113–177), were constructed into pE-SUMO vector (Figure 6b,c), with 19 overlapping amino acids among each other (Figure 6d).

### 3.4. Mapping of Linear B-Cell Epitopes on p22 Protein

**Figure 7:**
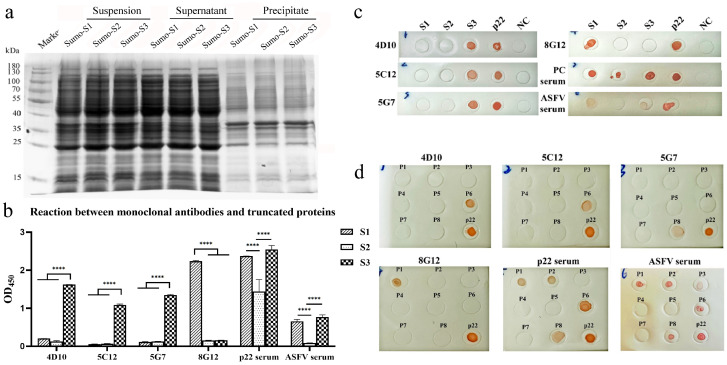
Mapping of Linear B-cell epitopes on p22 protein. (**a**) Identification of SUMO-fused truncated p22 protein by SDS-PAGE; (**b**) reaction between monoclonal antibodies and truncated p22 protein tested by ELISA. (The data has been shown as mean ± SD, **** *p* < 0.0001, n = 3); (**c**) reaction between monoclonal antibodies and truncated p22 protein tested by Dot-blot; (**d**) reaction between monoclonal antibodies and eight synthetic peptides of p22.

The truncated proteins were expressed in *E.coli Rossetta* (DE3) and identified by SDS-PAGE in Figure 7a. The supernatant after ultrasonication was used for antigen-coating. The results of ELISA (Figure 7b) and Dot-blot (Figure 7c) showed that S3 was detected by mAbs 4D10, 5C12 and 5G7, and S1 was detected by mAb 8G12. Both S1 and S3 could react with ASFV-positive pig serum, while S2 alone was detected by immunized mouse serum.

The truncated segments were further clipped into peptides for the epitope identification. The peptides with the sequences shown in Table 4 were conjugated with BSA in accordance with the protocol in 2.9. As is shown in Figure 7d, three linear epitopes were identified by mAbs. The results revealed that P6 (^140^WGTDDCTG^137^) was targeted by mAbs 4D10 and 5C12, which were sequenced to be the same variable region (Supplementary Figure S3). P8 (^150^YVYNNPHH^157^) was recognized by 5G7, and P1 (^30^KKQQPPKK^37^) was recognized by 8G12.

### 3.5. Conservation Analysis and Presentation of Linear B-Cell Epitopes on p22 Protein

**Figure 8:**
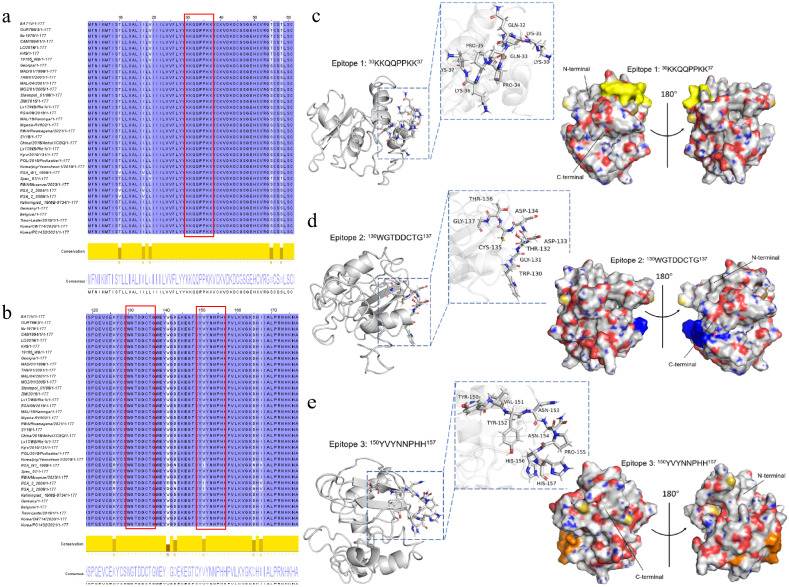
Conservation analysis and 3D model display of three linear B-cell epitopes on p22 protein. (**a**) Conservation analysis of epitopes ^30^KKQQPPKK^37^; (**b**) conservation analysis of epitopes ^130^WGTDDCTG^137^ and ^150^YVYNNPHH^157^; (**c**) three-dimensional model display of epitope ^30^KKQQPPKK^37^ by PyMOL software; (**d**) three-dimensional model display of epitope ^130^WGTDDCTG^137^ by PyMOL software; (**e**) three-dimensional model display of epitope ^150^YVYNNPHH^157^ by PyMOL software.

The conservation of the three B-cell epitopes of p22 protein identified above were analyzed in 36 isolates generated in Supplementary Table S1. According to the result of muscle alignment, the conservation of all three epitopes ^30^KKQQPPKK^37^, ^130^WGTDDCTG^137^, and ^150^YVYNNPHH^157^ in genotype I and genotype II epidemic strains were 100%. ^150^YVYNNPHH^157^ had the same mutation site I151 in genotype IV isolate RSA_W1_1999, genotype XX isolate RSA_2_2004 and genotype XXII isolate RSA_2_2008. Four mutation sites occurred in genotype VIII isolate NAM/91/2005, which are Y150, I151, Y156 and L157, respectively (Figure 8a,b).

The three B-cell epitopes were visualized on the dimensional structure model of the p22 protein predicted by I-TASSER modeling by PyMOL software (Version 3.0.3). The diagrams in Figure 8c–e illustrated that epitope 1 (^30^KKQQPPKK^37^) and epitope 2 (^130^WGTDDCTG^137^) were located in the loop downstream of the first and the last α-helix, and epitope 3 (^150^YVYNNPHH^157^) was located in the loop downstream of the last β-sheet.

## 4. Discussion

The major strategy of ASF control in affected countries relies primarily on systematic culling, which demands substantial financial and social resources over extended periods for eradication. Other integrated measures under OIE-recommended frameworks include active or passive monitoring, epidemiological investigations, proper disposal of infected carcasses and polluted wastes, stringent biosecurity controls for production facilities, and virus inactivation during transportation [30,31]. Beyond the complex ecology of natural ASFV hosts and limitations in laboratory diagnostics, the most critical barrier to ASF eradication is the absence of globally approved vaccines that are safe and effective. Current inactivated and attenuated live vaccines demonstrate suboptimal efficacy for entire protection for homologous and heterologous challenges accompanied by chronic infection or virulence reversal [32].

p22 has been demonstrated to play an important role in ASFV-associated multiple biological processes. The proteins interacting with p22 participate in endocytosis, DNA replication, spliceosome formation and the regulation of tRNA enzyme and mitochondrial function [33]. The structural and functional characteristics of p22 make it a prospective target for further research.

B-cell epitopes are broadly categorized into continuous (linear or sequential) and discontinuous (non-linear or conformational) types, spanning 6–20 and 20–400 amino acids, respectively. Current B-cell epitope identification relies on multiple techniques such as peptide scanning, site-directed mutagenesis [34], bacteriophage display [35,36], X-ray crystallography and cryogenic electron microscopy [37].

In recent years, the targets for B-cell epitope studies of ASFV have focused on the main structural proteins (including p72 [38,39], p54 [40], p30 [41,42], p17 [43,44] and CD2v [45]) and non-structural proteins (including pB602L [46], pE165R [47] and pK205R [48]). The main objective of epitope identification is to replace conventional whole antigens in antibody production and serodiagnosis by enhancing target specificity and reducing the risk of cross-reactivity [49]. Exploring and combining epitopes that are dominant or immune-protective during infection can facilitate the development of protective subunit vaccines against ASFV and the establishment of DIVA-compliant serological diagnostics [50]. Such approaches play important roles in the prevention of epidemics, monitoring infection status and tracing viral genetic evolution.

The cross-competition assay performed in this study was used to identify whether 11 hybridoma cell strains targeted the same epitope. By coating with a slight trace of antigen, the overlapping effect of antigen-binding between the saturated antibody and the other was detected. This method is often utilized for the identification of epitopes and the establishment of double-antibody sandwich ELISA [51,52]. Among those mAbs, no obvious competition was observed between 4D10 and 5C12. Subsequent sequencing confirmed that they indeed target distinct epitopes.

Notably, an epitope, P2 (^52^VRGSCSSLS^60^), that was not recognized by our mAbs reacted strongly with both immunized mouse serum and ASFV-positive pig serum. A weak reactivity was also observed in P3 (^66^KMDKRNIKIDSKIS^79^), which suggested that it might be a potential B-cell epitope inducing antibodies during immunization. The S2 segment was recognized by antiserum, but its reactivity was lost upon its truncation into short peptides, suggesting that S2 may constitute a conformational epitope.

The approaches used in this study, including bioinformatical methods and overlapping polypeptides design, are methodologically suitable and highly efficient for the initial broad-scale mapping of potential epitopes for interesting proteins, and provided a tractable and informative starting point for identifying conserved immunogenic regions across diverse viral strains. However, an inherent limitation of this approach is its inability to capture conformational or discontinuous epitopes. The determination of the high-resolution structure of the p22 protein alone and in combination with neutralizing monoclonal antibodies is our prospect for investigating conformational epitopes in future work. Furthermore, crucial follow-up work includes assessing the neutralizing activity of these epitopes at the cellular level and directly evaluating the protective efficacy of the induced immune response in animal models. This would involve determining if they can reduce disease severity, decrease viral replication, and increase survival rates upon challenge, thereby verifying their clinical value.

The recombinant *Escherichia coli* system has the advantages of high expression yield, low operating cost, clear genetic background and simple downstream purification, but has disadvantages such as the lack of post-translational modifications and the risk of endotoxin contamination (if intended for vaccine use). The expression of p22 in most studies at present was in the form of inclusion bodies. In this study, we achieved soluble expression to the largest extent possible as well as retaining sufficient immunological activity, demonstrating that the recombinant protein is highly suitable for use as a coating antigen for ASFV serological assays.

## 5. Conclusions

In this study, soluble recombinant ASFV p22 with high-yield production was prepared by a prokaryotic expression system. Following immunization with the purified antigen, eleven strains of mAb with high-affinity and titers were generated and characterized. Crucially, our comprehensive epitope mapping revealed three unreported linear B-cell epitopes: ^30^KKQQPPKK^37^, ^130^WGTDDCTG^137^ and ^150^YVYNNPHH^157^. These findings expanded the antigen spectrum of ASFV and enriched candidates for ASFV subunit vaccine formulation strategies.

## Figures and Tables

**Table 1:** PCR primers for amplification of truncated p22 protein.

Primer	Sequence of Nucleotide (5′→3′)
p22-F	CGCGGATCCAAGAAGCAGCAGCCTCCTAAGAAG
p22-R	CCGCTCGAGTTAGGCGTGCTTGTGGTTTCTAGG
S1-F	TTGGTCTCTAGGTAAGAAGCAGCAGCCTCCTAAGAAG
S1-R	CCGCTCGAGTTATCTGTAGAAGTTAGGGGTGAACTCAC
S2-F	TTGGTCTCTAGGTATCAAGATCGATTCTAAGATCTCTTCTTG
S2-R	CCGCTCGAGTTAGCCCCAAGAACAGTACTTCTCACAC
S3-F	TTGGTCTCTAGGTCCTTCTGAGTCTCACTCTCCTCAG
S3-R	CCGCTCGAGTTAGGCGTGCTTGTGGTTTCTAGG

**Table 2:** Orthogonal experiment for the optimization of expression conditions.

	Temperature for Expression (°C)	Concentration of IPTG (mM)	Incubation Time (h)
Group 1	16	0.2	8
Group 2	25	0.2	12
Group 3	37	0.2	16
Group 4	16	0.5	12
Group 5	25	0.5	16
Group 6	37	0.5	8
Group 7	16	0.8	16
Group 8	25	0.8	8
Group 9	37	0.8	12

**Table 3:** Integrated density of recombinant protein bands in orthogonal conditions analyzed by ImageJ.

	p22 Protein in Supernatant			p22 Protein in Precipitate			Ratio of Supernatant to Precipitate
	IntDen	Lane IntDen	Percentage (%)	IntDen	Lane IntDen	Percentage (%)	
Group 1	4.829	26.036	18.547	1.523	12.275	12.407	3.171
Group 2	5.198	30.261	17.177	1.265	11.234	11.260	4.109
Group 3	2.916	44.269	6.587	2.664	20.662	12.893	1.095
Group 4	8.315	44.837	18.545	3.528	20.007	17.634	2.357
Group 5	5.457	33.381	16.348	3.551	23.088	15.380	1.537
Group 6	4.634	54.733	8.467	6.721	38.989	17.238	0.689
Group 7	11.392	57.270	19.892	1.769	26.171	6.759	6.440
Group 8	7.397	44.687	16.553	2.599	28.571	9.097	2.846
Group 9	3.757	52.452	7.163	3.311	34.899	9.487	1.135

**Table 4:** Synthesis sequences of p22-peptides.

Name	Sequence of Amino Acid	Position
P1	CKKQQPPKK	30–37
P2	CVRGSCSSLS	52–60
P3	CKMDKRNIKIDSKIS	66–79
P4	CADEQQEFGKTRH	95–106
P5	CKITPSPSESHSP	109–120
P6	CWGTDDCTG	130–137
P7	CVGDEKEGT	141–148
P8	CYVYNNPHH	150–157

## Data Availability

The original contributions presented in this study are included in the article/Supplementary Material. Further inquiries can be directed to the corresponding author.

## Supplementary Materials

The following supporting information can be downloaded at: https://www.mdpi.com/article/10.3390/microorganisms13122666/s1, Figure S1: Standard curve of BSA for determination of the concentration of recombinant p22 protein and purified mAbs; Figure S2: Regression curve of affinity determination of monoclonal antibodies; Figure S3: Sequence of variable regions of monoclonal antibody; Table S1: ASFV isolates involved in multiple sequence alignment in this study.

## Abbreviations

The following abbreviations are used in this manuscript (alphabetical order):

DAPI	4′,6-Diamidino-2-phenylindole dihydrochloride
DIVA	differentiation of infected from vaccinated animals
DMSO	dimethylsulfoxide
eECL	enhanced Electrochemiluminescent
EDTA	ethylenediaminetetraacetic acid
ELISA	Enzyme-Linked Immunosorbent Assay
HRP	horseradish Peroxidase
IFA	Immunofluorescence Assay
IPTG	isopropyl β-D-thiogalactoside
PEG	polyethylene Glycol
PVDF	polyvinylidene fluoride
SDS-PAGE	sodium dodecyl sulfate polyacrylamide gel electrophoresis
SMCC	N-succinimidyl 4-(maleimidome)cyclo-hexanecarboxylate
