# Supplementary material for: Identification and Characterization of Three Novel B-Cell Epitopes in African Swine Fever Virus p22 Protein
Source: Microorganisms. 2025 Nov 24;13(12):2666. doi: 10.3390/microorganisms13122666 (PMC12735792; doi:10.3390/microorganisms13122666)
Supplement: Supplementary file 1 [file microorganisms-13-02666-s001.zip › microorganisms-3856885-supplementary.pdf]

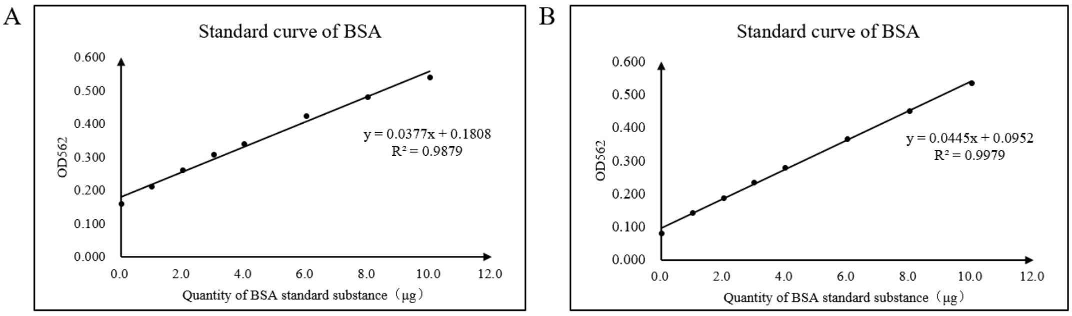

Supplementary figure S1. Standard curve of BSA for determination of the concentration of recombinant p22 protein and purified mAbs. (**A**) Standard curve of BSA for determination of the concentration of recombinant p22 protein; (**B**) Standard curve of BSA for determination of the concentration of purified mAbs.)

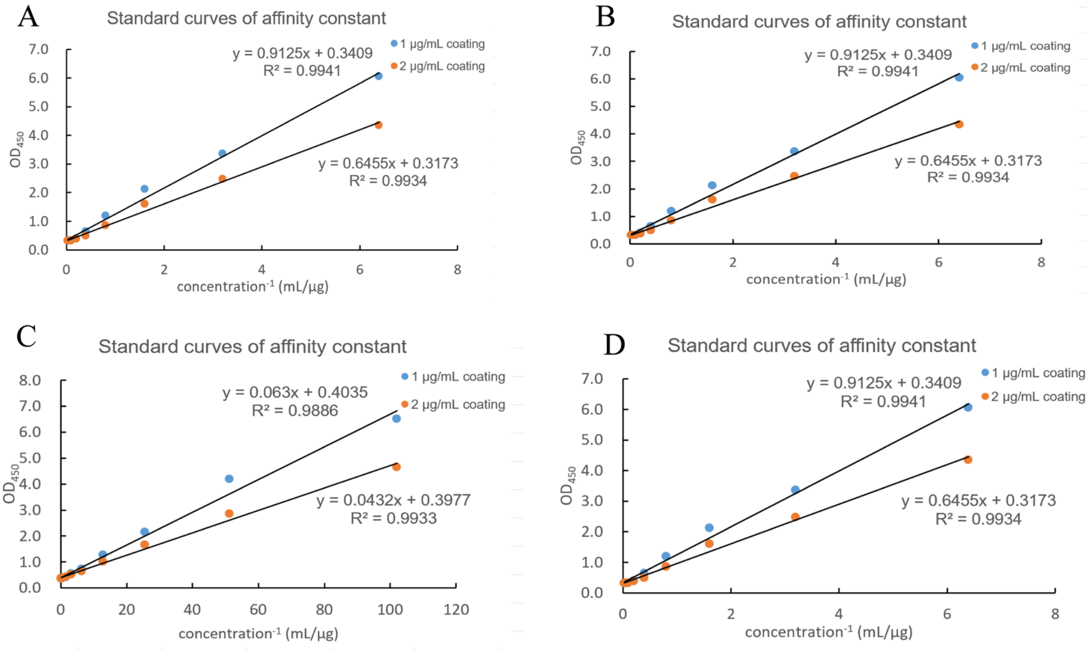

Supplementary figure S2. Regression curve of affinity determination of monoclonal antibodies. (**A**) mAbs 4D10; (**B**) mAbs 5C12; (**C**) mAbs 5G7; (**D**) mAbs 8G12.

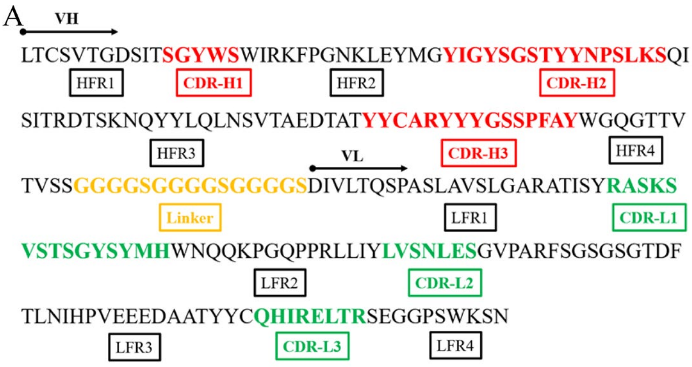

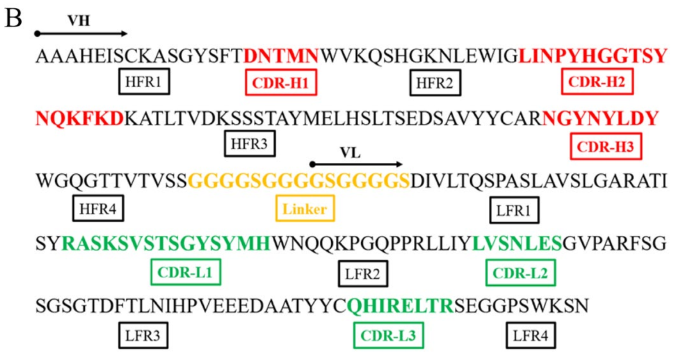

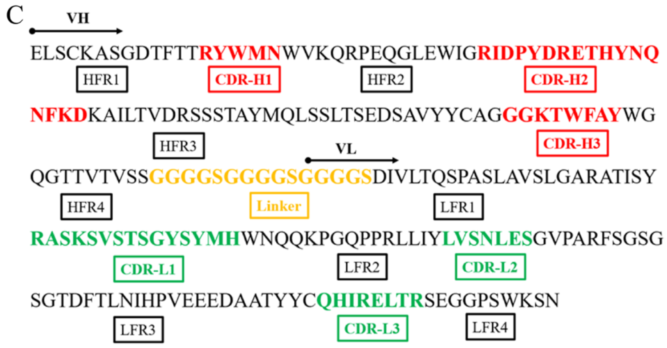

Supplementary figure S3. Sequence of variable regions of monoclonal antibody. (**A**) mAbs 4D10 and 5C12; (**B**) mAb 5G7; (**C**) mAb 8G12.

Supplementary table S1. ASFV isolates involved in multiple sequence alignment in this study

No.	Isolate	Country	Genotype	Genome Accession	Protein ID of p22
1	BA71V			NC_001659	NP_042704
2	OURT88/3	Portugal	I	NC_044957	YP_009703612
3	Nu1979	Italy	I	MW723481	UCX48452
4	CAM1994/1	Cameroon	I	OR387520	WNK21936
5	LO2018	Italy	I	MW647171	UFQ11170
6	K49	Zaire	I	MZ205520	QZK26688
7	19155_WB	Italy	I	OP312970	WFD50873
8	Georgia_2007/1	Georgia	II	NC_044959	YP_009927101
9	MAD/01/1998	Madagascar	II	OP781309	WLE29941
10	TAN/01/2001	Tanzania	II	QQ434234	WLE28849
11	MAL/04/2001	Malawi	II	OP781310	WLE30124
12	MOZ/01/2005	South Africa	II	OP781313	WLE30676
13	Stavropol_01/08	Russia	II	PQ672299	XNX60663
14	ZIM/2015	Zimbabwe	II	OP781311	WLE30308
15	Lv17/WB/Rie1i	Latvia	II	OR863252	WPS70783
16	RSA/08/2019	South Africa	II	OP781312	WLE30492
17	MAL/19/Karonga	Malawi	II	MW856068	QXP49940
18	Nigeria-RV502	Nigeria	II	OP672342	WFV29709

19	RWA/Rwamagana/2021	Rwanda	II	PQ375363	XOD29641
20	SY18	China	II	MH766894	WKD79495
21	China/2018/AnhuiXCGQ	China	II	MK128995	AYW33955
22	Lv17/WB/Rie1i	Spain	II	OR863252	WPS70783
23	Kyiv/2016/131	Ukraine	II	MN194591	QED21542
24	POL/2015/Podlaskie	Poland	II	MH681419	XBC37021
25	Korea/pig/Yeoncheon1/ 2019	Korea	II	MW049116	UID85690
26	RSA_W1_1999	South Africa	IV	MN641876	QST87078
27	Spec_57	South Africa	VIII	MN394630	UCX48452
28	RWA/Musanze/2023	Tanzania	IX	PQ375362	XOD29481
29	RSA_2_2004	South Africa	XX	MN641877	QII88854
30	RSA_2_2008	South Africa	XXII	MN336500	QGM12789
31	Kaliningrad_18/WB-9734	Russia	unannotated	OM966721	UVH35866
32	Germany 2020/1	Germany	unannotated	LR899193	CAD7112493
33	Belgium 2018/1	Belgium	unannotated	LR536725	VFV47910
34	Timor-Leste/2019/1	Australia	unannotated	MW396979	QTE18856
35	Korea/CW714/2020	Korea	unannotated	OR162436	WMM66466
36	Korea/PC1432/2021	Korea	unannotated	OR180305	WMQ58278
